# Highly Chlorinated Polyvinyl Chloride as a Novel Precursor for Fibrous Carbon Material

**DOI:** 10.3390/polym12020328

**Published:** 2020-02-05

**Authors:** Jinchang Liu, Hiroki Shimanoe, Seunghyun Ko, Hansong Lee, Chaehyun Jo, Jaewoong Lee, Seong-Hwa Hong, Hyunchul Lee, Young-Pyo Jeon, Koji Nakabayashi, Jin Miyawaki, Seong-Ho Yoon

**Affiliations:** 1 Interdisciplinary Graduate School of Engineering Sciences, Kyushu University, Kasuga, Fukuoka 816-8580, Japan; liujin.chang520@163.com (J.L.); 3ES17003K@s.kyushu-u.ac.jp (H.S.); nakabayashi@cm.kyushu-u.ac.jp (K.N.); miyawaki@cm.kyushu-u.ac.jp (J.M.); yoon@cm.kyushu-u.ac.jp (S.-H.Y.); 2 Carbon Industry Frontier Research Center, Korea Research Institute of Chemical Technology (KRICT), 141 Gajeong-ro Yuseong-gu, Daejeon 34114, Korea; feat@krict.re.kr; 3 Department of Fiber System Engineering, Yeungnam University, Gyeongsansi, Gyeongsanbokdo 38541, Korea; hansong.lee5@gmail.com (H.L.); jo1114@ynu.ac.kr (C.J.); jaewlee@yu.ac.kr (J.L.); 4 Korea Textile Machinery Research Institute (KOTMI), #27 Sampung-Ro, Kyungsan, Kyungsangbukdo 38542, Korea; shwhong@kotmi.re.kr; 5 Yusung Telecom Co. Ltd., 58-3, Yangdae-ro, Yangji-myeon, Cheoin-gu, Yongin-si, Gyeonggi-do 17158, Korea; Ihc1010@yusung82.co.kr; 6 University of Science and Technology (UST), 217 Gajeong-ro Yuseong-gu, Seoul 34113, Korea; 7 Institute for Materials Chemistry and Engineering, Kyushu University, Kasuga, Fukuoka 816-8580, Japan

**Keywords:** carbon fibre (CF), chlorinated polyvinyl chloride (CPVC), oxidative stabilisation, solid-state carbonisation

## Abstract

Pure, highly chlorinated polyvinyl chloride (CPVC), with a 63 wt % of chlorine, showed a unique-thermal-pyrolytic-phenomenon that meant it could be converted to carbon material through solid-phase carbonisation rather than liquid-phase carbonisation. The CPVC began to decompose at 270 °C, with a rapid loss in mass due to dehydrochlorination and novel aromatisation and polycondensation up to 400 °C. In this study, we attempted to prepare carbon fibre (CF) without oxidative stabilisation, using the aforementioned CPVC as a novel precursor. Through the processes of solution spinning and solid-state carbonisation, the spun CPVC fibre was directly converted to CF, with a carbonisation yield of 26.2 wt %. The CPVC-derived CF exhibited a relatively smooth surface; however, it still demonstrated a low mechanical performance. This was because the spun fibre was not stretched during the heat treatment. Tensile strength, Young’s modulus and elongation values of 590 ± 84 MPa, 50 ± 8 GPa, and 1.2 ± 0.2%, respectively, were obtained from the CPVC spun fibre, with an average diameter of 19.4 μm, following carbonisation at 1600 °C for 5 min.

## 1. Introduction

Carbon fibre (CF) is used for many industrial applications, e.g., automobiles, wind turbines and structural materials, which require high mechanical performance [1,2,3]. However, CF is still a relatively expensive engineering material in terms of its raw material costs. Additionally, CF manufacturing is also very extensive, requiring precursor preparation, spinning, oxidative stabilisation, carbonisation, graphitisation and surface treatment (sizing). Among the manufacturing processes, the most expensive step is considered to be the oxidative stabilisation step, because it is the most time- and energy-consuming process [4]. This step is where the spun precursor fibre is converted from the thermoplastic state into the thermosetting or infusible state through oxidative reactions. Decreasing the time and energy consumed by the oxidative stabilisation process by selecting a new oxidising agent or new precursor has been the long-time goal of numerous studies [4,5,6]. Otani et al. [6] developed a specially condensed polynuclear aromatic resin to reduce the oxidative stabilisation time. Considerable research has also been directed towards circumventing the oxidative stabilisation process by using special precursor polymers, such as poly(vinyl alcohol) [7], poly(brominated biphenyl) [8] and poly(benzimidazole) [9] as raw materials. However, the high cost of these polymer precursors [8,9], or the resulting low carbonisation yield and mechanical properties [7] of the obtained CFs, have proven to be insurmountable obstacles for implementation in CF applications.

Highly chlorinated polyvinyl chloride (CPVC), a typical low-cost polymer developed by excessive chlorination of common polyvinyl chloride (PVC), contains a higher amount of chlorine than common PVC. CPVC consists of three terpolymer units: 1,2-dichloroethylene (–CHClCHCl–), vinyl chloride (–CH_2_CHCl–) and 1,1-dichloroethylene (–CH_2_CCl_2_–) [10]. CPVC is commonly used as a structural material, or as part of a polymer blend, to enhance the flame-retardant and mechanical properties of products [11]. Compared with PVC, a greater number of pure conjugated aromatic hydrocarbons can be obtained by the thermal decomposition of CPVC owing to its chlorine content [12,13]. 

In contrast to common PVC decomposition, which occurs as a result of liquid-state carbonisation, CPVC decomposition can be attained through the solid-state carbonisation mechanism if the chlorinated amount is over 63 wt % [12,13]. Using this unique solid-state decomposition and the successive carbonisation of the CPVC, it may be possible to obtain well-defined, high-aromatic carbonaceous structures whilst maintaining form, only via heat treatment in an inert atmosphere without oxidative stabilisation. Yao et al. [14] successively obtained carbon nanofibre with a catalytic heat treatment in an inert atmosphere, using electrospun CPVC nanofibre as the precursor. However, so far it has been very difficult to use CPVC as a precursor for CF manufacturing, especially without any kind of oxidative stabilisation. It is almost impossible to obtain an adequate CPVC fibrous precursor for CF through melt spinning, because CPVC shows a very limited extension ratio, even if the chlorine atoms of CPVC are removed completely before melt spinning.

In this study, we first explored the potential of CPVC as a novel effective precursor for CF, specifically through only direct solid-state carbonisation in an inert atmosphere. Because CPVC is a relatively stiff polymer, the CPVC spun fibre was prepared using the wet solution spinning method. Although generally the cost efficiency of wet-type solution spinning is better than that of the melt spinning method, the efficiency of fibre production could be improved by increasing the number of spinnerets. In order to induce the fibre coagulation during the solution spinning process, the optimal preparation conditions were applied, determined by the analysis of coagulation behavior with the coagulation concentration. 

## 2. Materials and Methods 

### 2.1. Raw Material

Pure CPVC (H-17 grade; degree of polymerisation (DP), JIS K 6720-2; 750 ± 50; chloride content 63 wt %; Hanwha Chemical Co. Ltd., Seoul, Korea) was used without further treatment. The averaged molecular weights of *Mn* and *Mw*, which were determined by size-exclusion chromatography (SEC) with multiple detectors (online right-angle laser-light scattering and differential viscometric detectors [15]), were estimated as 51,975 and 113,165, respectively. The molecular weight distribution (*Mw/Mn*) was 2.177. 

*N*,*N*-Dimethylacetamide (DMAc) (Daejung Chemicals & Metals Co., Ltd., Gyeonggi-do, Korea) was used as a solvent for dissolving CPVC. Distilled water was used as a nonsolvent for the coagulation bath, in the wet-type solution spinning.

### 2.2. Preparation of CPVC Spun Fibre

**Figure 1:**
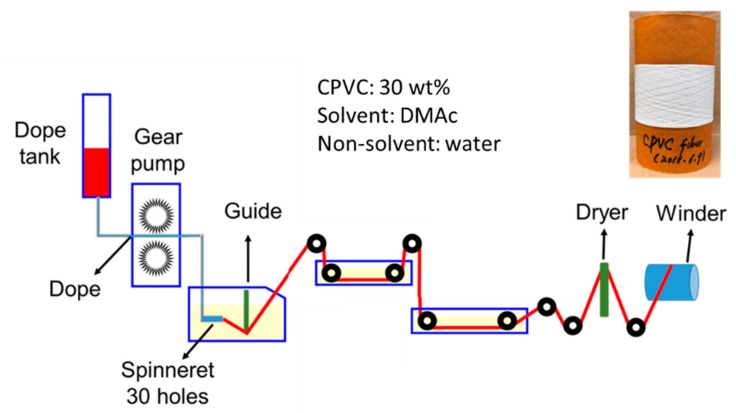
A schematic diagram of the self-designed apparatus for the solution spinning of highly chlorinated polyvinyl chloride (CPVC).

The CPVC spun fibre was obtained by solution spinning using a custom-designed spinning apparatus, as shown in Figure 1. The prepared CPVC solution at the ambient temperature was discharged at the pressure of 0.6 MPa, with the rate of gear pump of 0.475 mL/min, and discharged into a solidifying bath through 30-hole spinnerets with a diameter of 0.1 mm. To solidify the fibrous CPVC discharged solution, the speed of the solvent exiting the fibre was controlled by mixing DMAc (solvent) and water (nonsolvent) at a volume ratio of 70/30, and the temperature of the solidifying solution was also adjusted to 30 °C. The coagulated, fibrous CPVC was washed twice with distilled water to remove any remaining solvent. After washing, the fibrous CPVC was dried by passing it through a heat chamber (dryer) at 100 °C to ensure an easy separation of the CPVC fibres and maintain their round shape, and it was wound with a winding speed of 6.55–7.05 m/min. After winding, the CPVC fibres were immersed in distilled water at 30 °C and dried naturally at room temperature for 24 h to produce the CPVC spun fibre. They were then dried again at 120 °C for 5 h and under vacuum, to ensure the complete removal of any remaining solvent. The final fibrous CPVC precursor obtained showed an average diameter of 54 ± 4.2 μm. To determine the average diameter of the fibrous CPVC, 200 samples were observed under SEM. 

### 2.3. Preparation of a CPVC-Derived Carbon Fibre

The CPVC spun fibres (as described above) were then directly heat-treated for carbonisation from 100 to 1000 °C, under 100 mL/min of N_2_ flow. The heating rate was carefully controlled as follows: (1) 1 °C/min from 100–450 °C, (2) 5 °C/min from 450–1000 °C and (3) held for 5 min after reaching 1000 °C. To improve the mechanical properties of the CPVC fibres, the carbonised fibres were further heat-treated at 1600 °C in a vacuum [16]. 

### 2.4. Characterisation

The molecular weight, and its distribution in the CPVC sample, were characterised using SEC with multiple detectors: online right-angle laser-light scattering and differential viscometric detectors. The chromatography parameters of the sample CPVC were determined using a high-performance size-exclusion chromatography (HPSEC), the Viscotek 270 dual detector (Viscotek, Houston, TX, USA) with a differential viscometer (DV), a right-angle laser-light scattering detector (RALLS; Viscotek, Houston, TX, USA) and a refractive index (RI) (Knauer K2301) detector. The column set consisted of a HPLC 10 lm guard column (50 mm·7.5 mm), followed by two GMH_XL_-L HPLC columns (300 mm·7.5 mm, 10 lm, Tosoh, Japan). Equipped with a HPLC pump (Knauer K-1001) set with a flow rate of 1 mL/min, the eluent was previously filtered through a 0.2 lm filter. The system was equipped with a Knauer on-line degasser. The tests were conducted at two different temperatures (25 and 30 °C) using an Elder CH-150 heater. Before the injection (100 ll), the samples were filtered through a poly(tetrafluoroethylene) (PTFE) membrane with 0.2 lm pores. The system was calibrated following narrow polystyrene standards. The differential RI (dn/dc) used for 670 nm was determined as 0.105. The analysis of the light scattering data by software was completed assuming the second virial coefficient was zero, considering the low solution concentrations used in this work. The parameters studied in this work (*Mz*, *Mw*, *Mn*, *g*, *Rg* and *Rh*), were calculated using the software provided by Viscotek (version 3.0). It should also be noted that the elution times of the RALLS, DV, and RI detectors and the volume calculation according to the RI detector were adjusted by using the software. The CPVC samples of 0.250 ± 0.002 g were weighed and added to 100 mL volumetric flasks. The flasks were filled 2/3 of the volume with tetrahydrofuran (THF) and carefully stirred in a temperature-controlled glycerin heating bath at 85 ± 5 °C, until complete dissolution. After that, the solutions were allowed to cool down to room temperature. The flasks were then filled completely with cyclohexanone preheated at 85 ± 5 °C for 90 min and placed in an isotherm bath at 30 ± 0.5 °C for 20 min. The THF volume was readjusted for the solvent at 30 ± 0.5 °C. The sample was then stirred until complete dissolution and filtered through a porous plaque G-1. Using an AVS 50 viscometer, the time constant for the solvent (*t*_0_) and the samples were determined (*t*). To validate the sample measurements, three determinations were carried out for each sample, considering the maximum admissible difference to be 0.1%.

The pyrolytic decomposition properties of the CPVC, PVC (the degree of polymerisation was approximately 1100; Wako Pure Chemical Industries, Ltd., Osaka, Japan) and CPVC spun fibres were measured by thermogravimetric analysis (TGA) (SII 6300 Exstar; Seiko Co. Ltd., Tokyo, Japan). TGA measurements of PVC and CPVC were carried out using 8 mg of each sample, under nitrogen flow, from room temperature (RT) to 1000 °C. The amount of nitrogen was controlled at 200 mL/min, and the heating rate was set to 5 °C/min. The TGA measurement of the CPVC spun fibre was also carried out from RT to 350 °C, using two heating rates (3 and 5 °C/min) under 100 mL/min of air flow to track the weight gain of oxygen.

Solid-state ^13^C nuclear magnetic resonance spectroscopy (^13^C-NMR) (ECA400; JEOL Co. Ltd., Akishima, Japan; 400 MHz) was used to investigate the quantitative changes in the molecular structures of the PVC, CPVC and CPVC spun fibres, after heat treatment at 200, 300, 400 and 1000 °C, under an argon atmosphere. The chemical shift calibration was based on the methyl carbon resonance of solid hexa-methyl benzene at a chemical shift of 17.4 ppm. About 100 mg of the pulverised sample was placed in a standard zirconia sample rotor (diameter: 3.2 mm). Spectra were acquired at room temperature with 4026 scans. The acquisition time was 0.05 s, with a pulse of 90° and a spectrum width of 15 kHz. The method of ^13^C detection was DEPTH2, with a magic-angle spinning speed of 15 kHz.

Scanning electron microscopy (SEM) (JSM-6700F; JEOL Co. Ltd., Akishima, Japan) was used to examine the fibre morphology of the CPVC spun fibres (coated by 20 nm of OsO_4_) and the carbonised CPVC fibres. SEM images were acquired with an accelerating voltage of 10 kV, at different magnifications.

The tensile strength, the Young’s modulus and the elongation property of the obtained CPVC-derived CFs were measured according to the JIS R 7601 (a testing method for CFs, based on Japanese industrial standards), with a strength testing apparatus (Tensilon UTM-11-20; Orientec Co. Ltd., Tokyo, Japan). A laser measuring instrument (M550A; Anritsu Devices Co. Ltd., Kanagawa, Japan) was used to check the fibre diameter of 20 samples. The mechanical properties of the CPVC-derived CF monofilaments were measured 20 times to reduce error, and the average of the data was reported.

## 3. Results

### 3.1. The Pyrolytic Behaviours of PVC and CPVC 

**Figure 2:**
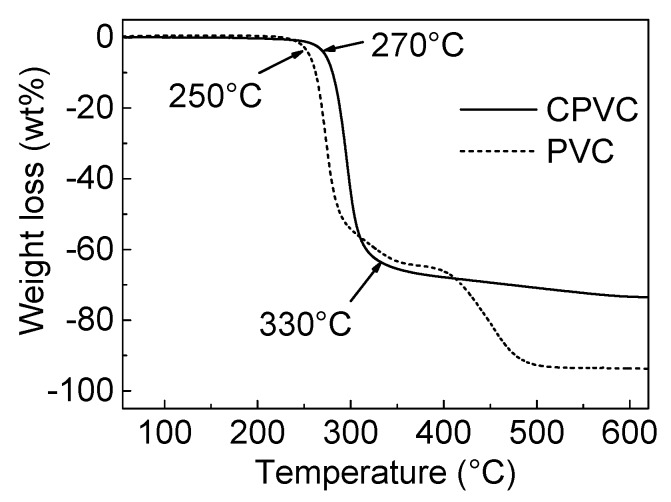
The thermogravimetric analysis (TGA) profiles of the chlorinated polyvinyl chloride (CPVC) and polyvinyl chloride (PVC) under N_2_ flow.

Figure 2 shows the pyrolytic decomposition behaviors of the PVC and CPVC via TGA. PVC was thermally stable up to 250 °C, at which point decomposition was initiated [15,17]. In the temperature range of 250–330 °C, rapid dehydrochlorination and formation of polyene-type molecules occurred [15,17,18]. The mass loss of PVC at this stage was ~56 wt %. In the temperature range of 330–450 °C, the remaining polyene-type molecules were converted into polycondensed aromatic hydrocarbons and eventually into infusible coke through liquid carbonisation [15,17]. PVC showed around 9 wt % fixed carbon yield after heat treatment at 900 °C for 0.5 h. 

The CPVC displayed unique pyrolytic decomposition behavior when compared with PVC. As the TGA curves show in Figure 2, the CPVC remained in a thermally stable state up to 270 °C, which was 20 °C higher than the PVC. The CPVC started to decompose from 270 °C, due to the reaction of dehydrochlorination. In the temperature range of 330–590 °C, the remaining polyene and polyene-type molecules underwent a gradual weight reduction. The CPVC showed 26.7 wt % fixed carbon yield after heat treatment at 900 °C for 0.5 h; TGA and DTG were scanned up to 1000 °C, and the complete profiles are listed in Figure S1 in the Supplementary Materials. The produced polyene and polyyne-type molecules from the CPVC decomposition were converted into polycondensed aromatic molecules, and finally into coke, through solid-state carbonisation.

**Figure 3:**
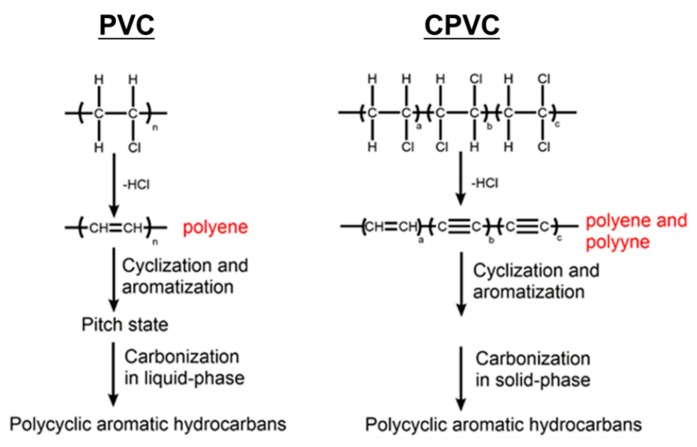
The conjectured mechanisms of decomposition and carbonisation of the PVC and CPVC.

Regarding the TGA analysis of the PVC and the CPVC, the results are summarised as follows:

(1) The PVC decomposition was initiated with dehydrochlorination, resulting in a polyene-type molecule formation over the temperature range of 250–330 °C. These molecules were then converted into polycondensed aromatic compounds, through liquid-state carbonisation, over the temperature range of 310–400 °C. From there, the molecules entered an infusible state (i.e., coke formation) in the range of 400–480 °C before being converted into a carbon material after heat treatment above 650 °C. 

(2) The CPVC started to decompose with the dehydrochlorination and formed polyene and polyene-type molecules in the temperature range of 250–330 °C. These molecules were then converted into infusible three-dimensional cross-linked polycondensed aromatic compounds through solid-state carbonization from 310–650 °C, as opposed to the decomposition and carbonisation processes of the PVC. The compounds were finally converted into carbon material after heat treatment at temperatures above 650 °C.

(3) The PVC carbonisation occurred via the liquid-phase carbonisation, whereas the CPVC carbonisation took place in the solid phase. The flowcharts for the PVC carbonisation and the CPVC carbonisation are shown in Figure 3.

**Figure 4:**
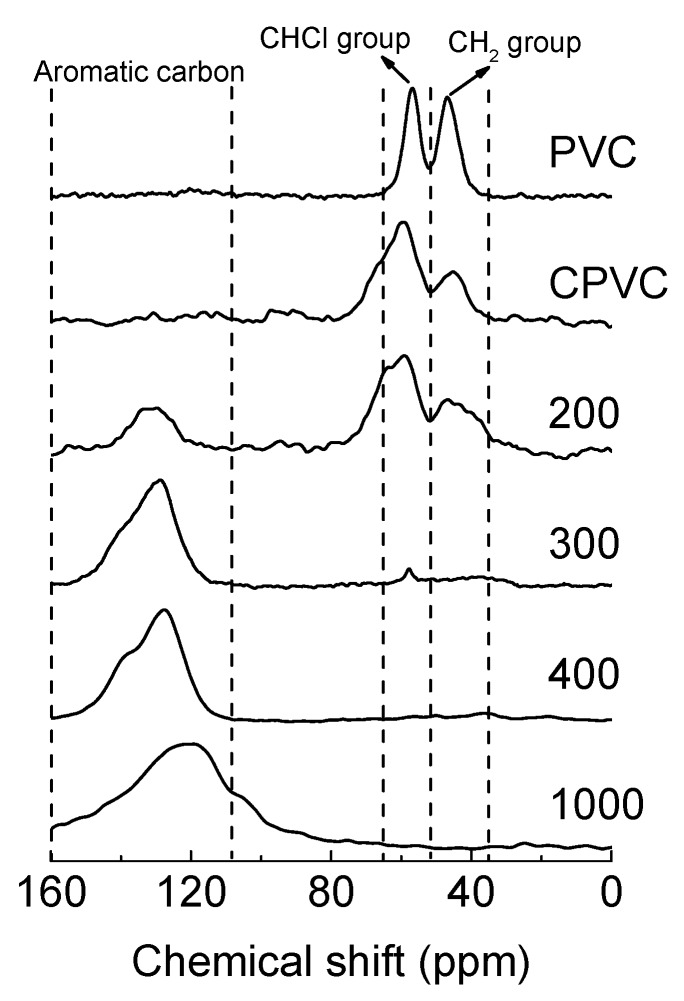
The solid-state ^13^C nuclear magnetic resonance spectroscopy (^13^C-NMR) spectra of the PVC, CPVC and carbonised intermediates of the CPVC spun fibres, at 200, 300 and 400 °C, and the CPVC carbon fibre heat treated at 1000 °C.

Figure 4 shows the ^13^C-NMR spectra of the CPVC and its pyrolysed intermediates after heat treatment at various temperatures (200, 300, 400 and 1000 °C). Table 1 summarises the molecular structures and yields of the intermediates and carbonised samples. The chlorinated carbon groups of the CPVC, –CHCl– and –CCl_2_– had a higher weight percentage than those of PVC, showing values of 74.0 and 48.6 wt %, respectively. Due to the more disordered molecular structure of CPVC and the quadruple interaction of ^35^Cl and ^37^Cl nuclei [19], the peak width of CPVC was wider than that of PVC. At a carbonised temperature of 200 °C, the chlorinated carbon groups and methylene groups were converted into aromatic molecules, and the amount of chlorinated carbon molecules decreased by ~20 wt %. Almost all C-Cl bonds were broken at temperatures above 300 °C, and the aromatic molecule content increased to 96.2 wt %. The characteristic peak of chlorinated molecules disappeared as the heat treatment temperature exceeded 400 °C. 

Interestingly, as shown in ^13^C-NMR results of Figure 4, the aromatic molecules began to form at 200 °C and almost all of the molecules of –CHCl– and –CHC– were converted into aromatic molecules at 300 °C in the solid state. Additionally, the formed aromatic molecules were also converted to polyaromatic molecules at 400 °C. Most carbon groups of the CPVC fibres were converted into carbon material after heat treatment at 1000 °C, in which the aromatic molecule content reached 99.2 wt %.

**Figure 5:**
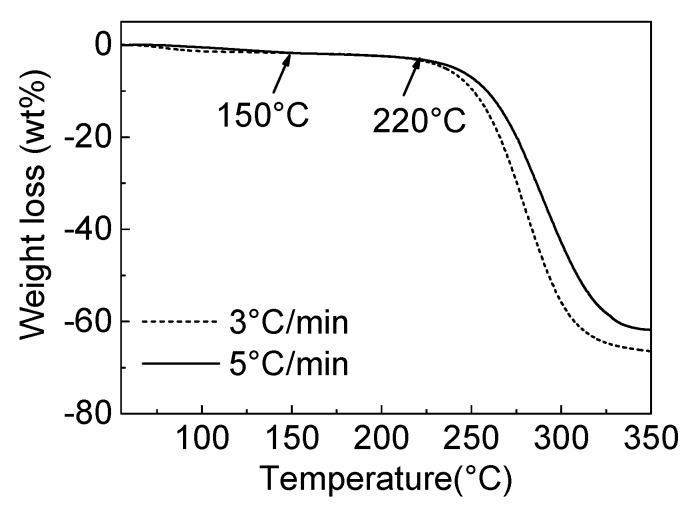
The TGA profiles of the CPVC spun fibres, under air flow with different heating rates.

The oxidation pyrolytic decomposition of the CPVC spun fibre was analysed by the TGA, as shown in Figure 5. The primary weight loss occurred between 220 and 350 °C, regardless of the heating rate, which is similar to the the TGA results of the CPVC under a nitrogen atmosphere, as shown in Figure 2. From this result, we confirmed the CPVC decomposition and conversion to carbon material through solid-state carbonisation. 

### 3.2. The Scanning Electron Microscope (SEM) Images and the Mechanical Properties of the CPVC-Derived Carbon Fibres (CFs)

**Figure 6:**
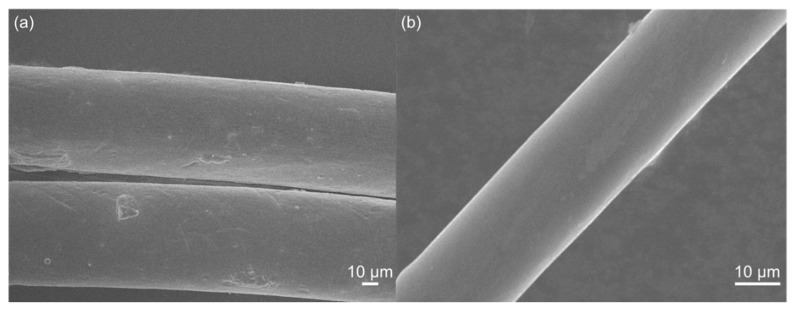
The scanning electron microscopy (SEM) micrographs of (**a**) CPVC spun fibre and (**b**) the CPVC-derived carbon fibre heat-treated at 1000 °C.

Figure 6 shows SEM images of the CPVC-spun fibres and the CPVC-derived CFs. The obtained CFs exhibited very smooth surfaces, almost no discernable defects, and a diameter that was considerably smaller than that of the CPVC spun fibres. The diameter of the CPVC CFs was around 20 μm, due to the shrinkage that occurred during carbonisation. The surface of the CPVC CFs was homogenous and smooth, although a slight trace was observed. In the cross-sectional view, a smooth and uniform microstructure was observed, as shown in Figure S2 in the Supplementary Materials.

Table 2 shows the average diameter and mechanical properties of the CPVC CFs. Using a carbonised temperature of 1000 °C, the average diameter of the CPVC CFs was 20.4 μm. The tensile strength, Young’s modulus and elongation were 480 ± 58 MPa, 39 ± 6 GPa and 1.3 ± 0.2%, respectively. When the CPVC spun fibres were successively heat-treated at 1600 °C, the tensile strength, Young’s modulus and elongation improved to 590 ± 84 MPa, 49 ± 8 GPa and 1.2 ± 0.2%, respectively, with an average diameter of 19.4 μm. The number of cumulative carbon layers and the length of the average carbon layer were increased as the carbonisation temperature increased [16]; therefore, the mechanical properties were enhanced when the temperature increased from 1000 to 1600 °C. The tensile strength that the CPVC CF showed (590 MPa) is quite comparable with those of the isotropic pitch-derived carbon fibres (IPCFs) having similar diameters: Ko et al. [20] reported that an IPCF with an average diameter of 18.5 μm showed 470 MPa in tensile strength, and Park et al. [21] reported IPCFs with diameters of 19.0 and 19.3 μm showed 581 and 529 MPa, respectively. Currently, those IPCFs with a tensile strength less than 800 MPa (a Young’s modulus of 30–50 GPa) are mostly used for activated carbon fibres. The CPVC CFs that show similar mechanical performances with IPCFs are thus thought to be applicable to activated carbon fibre at this stage. Moreover, if its mechanical performance is further improved to 1.7 and 170 GPa in tensile strength and Young’s modulus, respectively, it can also be applicable to car body and windmill structure [22], because the CPVC CF would have high advantages in terms of production costs, since its materials are low-cost and it has capability of skipping over the oxidative stabilisation process, which is the most costly step of carbon fibre production. This is going to be a very challenging task, but we consider it to be achievable and our group is currently conducting related studies like reducing fibre diameter to half the current size and modification of the molecular arrangement and molecular weight distribution of the CPVC.

## 4. Conclusions

The CF was successfully prepared without oxidative stabilisation using the CPVC as a novel precursor. The spun fibres of the CPVC, which contained 63 wt % chlorine, were converted into CF with a carbonisation yield of 26.2 wt % through solid-state carbonisation after heat treatment at 1000 °C for 5 min. The CPVC decomposed at 270 °C with a rapid mass loss due to dehydrochlorination, and the novel formation of aromatisation and polycondensation occurred up to 400 °C. The obtained CPVC-derived CFs exhibited smooth surfaces with relatively low mechanical performance prior to stretching the spun fibres during the carbonisation process. Under carbonisation at 1600 °C, the tensile strength, the Young’s modulus and the elongation property of the CPVC CFs were 590 ± 84 MPa, 49 ± 8 GPa and 1.2 ± 0.2%, respectively, with an average fibre diameter of 19.4 μm.

## Figures and Tables

**Table 1:** The yields and molecular structures of the PVC, CPVC and thermally decomposed intermediates of the CPVC spun fibres and the CPVC-derived carbon fibre.

Sample ^1^	Yield (wt %)	The Amount of Carbon Groups ^2^		
		Aromatic Carbon (wt %) ^3^	Aliphatic Carbon (wt %) ^4^	
			Chlorinated Carbon (–CCl_2_–, –CHCl–)	Methylene Carbon (–CH_2_–)
PVC	--	--	48.6	51.4
CPVC	--	--	74.0	26.0
200	38.3	21.2	54.2	24.6
300	29.7	96.2	1.9	1.9
400	28.0	98.8	~0	1.2
1000	26.2	99.2	0	0.8

^1^ The samples for ^13^C-NMR detection, including the PVC, CPVC and carbonised intermediates of the CPVC fibres, at 200, 300 and 400 °C, and the CPVC carbon fibres treated at 1000 °C. ^2^ The amount of carbon groups were calculated by integrating peak areas on ^13^C NMR spectra. ^3^ Aromatic carbon: 108–160 ppm. ^4^ Aliphatic carbon: 17–80 ppm [3].

**Table 2:** The mechanical properties of the CPVC derived carbon fibres.

CPVC Derived Carbon Fibres ^1^	Diameter (μm)	Tensile Strength (MPa)	Young’s Modulus (GPa)	Elongation (%)
1000 °C	20.4 ± 1.8	480 ± 58	39 ± 6	1.3 ± 0.2
1600 °C	19.4 ± 2.3	590 ± 84	49 ± 8	1.2 ± 0.2

^1^ CPVC carbon fibres carbonised at 1000 °C and 1600 °C, respectively.

## Supplementary Materials

The following are available online at https://www.mdpi.com/2073-4360/12/2/328/s1, Figure S1. Thermo-gravimetric analysis (TGA) profiles of chlorinated polyvinyl chloride (CPVC) and polyvinyl chloride (PVC) under N_2_ flow, showing weight loss (left y-axis) and differential curves of the weight loss (right y-axis) from ambient temperature to 1000 °C; Figure S2. Scanning electron microscopy (SEM) micrographs of (a) lateral and (b) cross-sectional views of the CPVC derived carbon fibers.
