# Supplementary material for: Highly Chlorinated Polyvinyl Chloride as a Novel Precursor for Fibrous Carbon Material
Source: Polymers (Basel). 2020 Feb 5;12(2):328. doi: 10.3390/polym12020328 (PMC7077477; doi:10.3390/polym12020328)
Supplement: Supplementary file 1 [file polymers-12-00328-s001.pdf]

Supplement Information for

Highly chlorinated polyvinyl chloride as a novel precursor for fibrous carbon material

Jinchang Liu[1], Hiroki Shimanoe[1], Seunghyun Ko[2], Hansong Lee[3], Caehyun Jo[3], Jaewoong Lee[3], Seong-Hwa Hong[4], Hyunchul Lee[5], Young-Pyo Jeon[2,6,*], Koji Nakabayashi [1,7], Jin Miyawaki [1,7], and Seong-Ho Yoon [1,7]

1 Interdisciplinary Graduate School of Engineering Sciences, Kyushu University, Kasuga, Fukuoka, 816-8580, Japan; liujin.chang520@163.com (J. Liu); 3ES17003K@s.kyushu-u.ac.jp (H. Shimanoe); nakabayashi@cm.kyushu-u.ac.jp (K. Nakabayashi); miyawaki@cm.kyushu-u.ac.jp (J. Miyawaki); yoon@cm.kyushu-u.ac.jp (S.-H. Yoon)

2 Carbon Industry Frontier Research Center, Korea Research Institute of Chemical Technology (KRICT), 141 Gajeong-ro Yuseong-gu, Daejeon 34114, Korea; feat@krict.re.kr (S. Ko); ypjeon@krict.re.kr (Y.-P. Jeon)

3 Department of Fiber System Engineering, Yeungnam University, Gyeongsansi, Gyeongsanbokdo 38541, Korea; hansong.lee5@gmail.com (H. Lee); jo1114@ynu.ac.kr (C. Jo); jaewlee@yu.ac.kr (J. Lee)

4 Korea Textile Machinery Research Institute (KOTMI), #27 Sampung-Ro, Kyungsan, Kyungsangbukdo 38542, Korea; shwhong@kotmi.re.kr (S.-H. Hong)

5 Yusung Telecom Co. LTD, 58-3, Yangdae-ro, Yangji-myeon, Cheoin-gu, Yongin-si, Gyeonggi-do 17158, Korea; Ihc1010@yusung82.co.kr (H. Lee)

6 University of Science and Technology (UST), 217 Gajeong-ro Yuseong-gu, Daejeon 34113, Korea

7 Institute for Materials Chemistry and Engineering, Kyushu University, Kasuga, Fukuoka, 816-8580, Japan

* Correspondence: ypjeon@krict.re.kr; Tel.: +82-42-860-7199

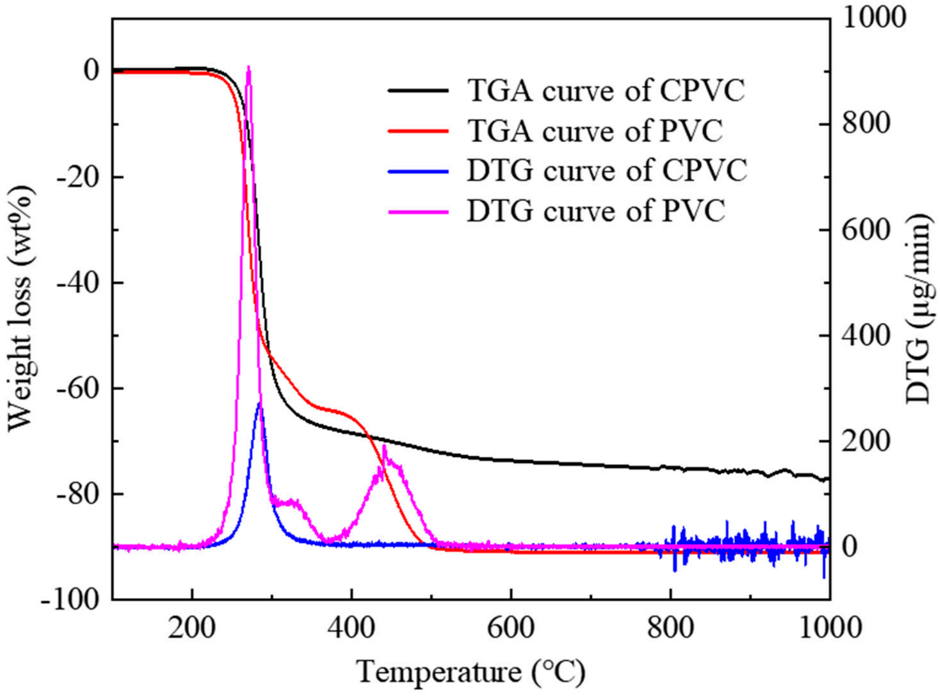

Figure S1. Thermo-gravimetric analysis (TGA) profiles of chlorinated polyvinyl chloride (CPVC) and polyvinyl chloride (PVC) under N_2 flow, showing weight loss (left y-axis) and differential curves of the weight loss (right y-axis) from ambient temperature to 1,000 ˚C.

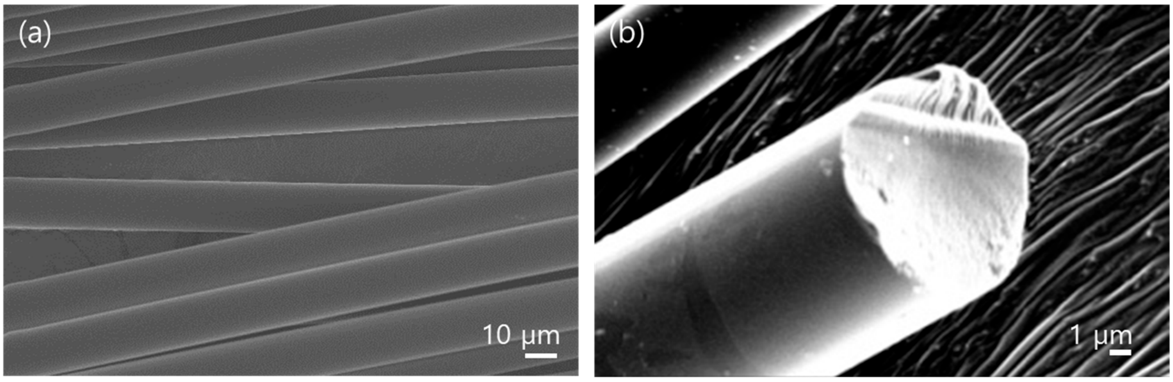

Figure S2. Scanning electron microscopy (SEM) micrographs of (a) lateral and (b) cross-sectional views of the CPVC derived carbon fibers.
